# Peripheral injections of melanin-concentrating hormone receptor 1 antagonist S38151 decrease food intake and body weight in rodent obesity models

**DOI:** 10.3389/fendo.2012.00160

**Published:** 2012-12-21

**Authors:** Odile Della-Zuana, Valérie Audinot, Viviane Levenez, Alain Ktorza, Françoise Presse, Jean-Louis Nahon, Jean A. Boutin

**Affiliations:** ^1^Maladies Métaboliques, Institut de Recherches SERVIERSuresnes, France; ^2^Biotechnologie, Pharmacologie Moléculaire et Cellulaire, Institut de Recherches SERVIERCroissy-sur-Seine, France; ^3^Genomics and Evolution in Neuroendocrinology, Institut de Pharmacologie Moléculaire et Cellulaire, UMR7275, Centre National de la Recherche ScientifiqueValbonne, France; ^4^Genomics and Evolution in Neuroendocrinology, Université de Nice Sophia AntipolisNice, France

**Keywords:** melanin-concentrating hormone, receptors, peptide antagonists, feeding behavior, rat, mice, obesity models

## Abstract

The compound S38151 is a nanomolar antagonist that acts at the melanin-concentrating hormone receptor 1 (MCH_1_). S38151 is more stable than its purely peptide counterpart, essentially because of the blockade of its N-terminus. Therefore, its action on various models of obesity was studied. Acute intra-cerebroventricular (i.c.v.) administration of S38151 in wild-type rats counteracted the effect of the stable precursor of melanin-concentrating hormone (MCH), NEI-MCH, in a dose-dependent manner (from 0.5 to 50 nmol/kg). In genetically obese Zucker *fa/fa* rats, daily i.c.v. administration of S38151 induced dose-dependent (5, 10, and 20 nmol/kg) inhibition of food intake, water intake, and body weight gain, as well as increased motility (maximal effect observed at 20 nmol/kg). In Zucker *fa/fa* rats, intraperitoneal injection of S38151 (30 mg/kg) induced complete inhibition of food consumption within 1 h. Daily intraperitoneal injection of S38151 (10 and 30 mg/kg) into genetically obese *ob/ob* mice or diet-induced obese mice is able to limit body weight gain. Furthermore, S38151 administration (10 and 30 mg/kg) does not affect food intake, water intake, or body weight gain in MCHR1-deleted mice, demonstrating that its effects are linked to its interaction with MCH_1_. These results validate MCH_1_ as a target of interest in obesity. S38151 cannot progress to the clinical phase because it is still too poorly stable *in vivo*.

## Introduction

Melanin-concentrating hormone (MCH) is a 17-amino-acid, cyclic pseudopeptide that is responsible for the bleaching of skin in teleost fishes (Kawauchi et al., 1983). In mammals, MCH is a 19-amino-acid cyclic peptide (Vaughan et al., 1989). Its actions are not linked to pigmentation, but to a variety of properties, originally centered on appetite regulation and obesity (Qu et al., 1996; Pissios et al., 2006). MCH acts in several processes, including sleep/arousal (Hervieu, 2006; Peyron et al., 2009), emotionality (Hervieu, 2006), and memory (Adamantidis and de Lecea, 2009). MCH is encoded as a pre-pro-hormone, and also gives rise to two other neuropeptides after proteo-cleavage: (1) neuropeptide N-G (NGE), the function of which is elusive, and (2) neuropeptide E-I (NEI) (Nahon et al., 1989), which displays various functions (Maulon-Feraille et al., 2002; Bittencourt and Celis, 2008). NEI-MCH is more potent than MCH in stimulating feeding in rats, for proteolytic-protective reasons (Maulon-Feraille et al., 2002). MCH is mainly synthesized in cell bodies of the lateral hypothalamus and sub-*zona incerta* in the central nervous system (Bittencourt et al., 1992). MCH is also synthesized in peripheral organs, such as the gut, arteries, testes, thymus, and pancreatic β-cells (Hervieu and Nahon, 1995).

Two MCH receptors (MCH_1_ and MCH_2_) were identified a decade ago. MCH_1_ was known for quite some time as SLC-1, an orphan receptor in humans (Lakaye et al., 1998). It was established as a genuine MCH receptor in 1999 by at least three different groups (Bachner et al., 1999; Chambers et al., 1999; Saito et al., 1999). The subsequent search for analogs of the protein led to the cloning of a second receptor, MCH_2_ (Mori et al., 2001; Rodriguez et al., 2001; Sailer et al., 2001; Wang et al., 2001). This second receptor is expressed in humans, dogs, and ferrets, but not in rodents. In mammalian species, the highest expression levels of both receptors are found in the frontal cortex, amygdala, and nucleus accumbens; however, they are also expressed in hypothalamic areas regulating energy balance, such as the arcuate nucleus and the ventral medial hypothalamus. MHCRs are also moderately expressed in peripheral organs. In rodents, MCH acts through MCH_1_, which implicates MCH_1_ in obesity and energy homeostasis.

Several genetically engineered strains of mice have been reported. MCH_1_-knockout (MCH_1_-KO) mice (mice do not express a functional MCHR_2_ protein) exhibit a phenotype of leanness and resistance to diet-induced obesity, characterized by hyperphagia, hyperactivity, and hypermetabolism (Chen et al., 2002; Marsh et al., 2002; Pissios, 2009). This phenotype is attributed to increased energy expenditure, resulting from increased locomotor activity and increased resting energy expenditure. Paradoxically, these mice exhibit significant hyperphagia.

The molecular pharmacology of MCH_1_ has been studied in some detail. After the centrally important works of teams at Takeda and Synaptic/Lundbeck (Borowsky et al., 2002; Takekawa et al., 2002), many non-peptide compounds were screened and found active at MCHR1. Most of these small molecules inhibit MCH-mediated feeding behavior; however, they may also affect energy expenditure. Interestingly, MCH_1_ antagonists of different chemical classes exhibit anxiolytic and anti-depressant effects, a finding that would likely contribute to the success of these molecules in obese populations that might also have depression and anxiety. However, cardiovascular risk associated with human ether-a-go-go-related gene (hERG)-binding activity of biaryl-containing compounds has plagued many of these non-peptide MCH_1_ antagonist programs (Mendez-Andino and Wos, 2007; Meyers et al., 2007; Johansson, 2011).

Alternatively, several dozen MCH analogs have been synthesized and tested by binding, leading to the discovery of peptide super-agonists and mild antagonists. Some of these have been tested on MCH_2_, and present major selectivity to MCH_1_. Structure activity relationship is now well-established concerning the peptide ligands. Although small molecules had been synthesized as potent and moderately selective MCH_1_ antagonists, there was still an interest in finding new peptides with antagonistic activities. Pioneering works by Bednarek's group described such interesting compounds. In agreement with our initial search for better agonists (Audinot et al., 2001a,b), we explored the possibility of designing natural peptides or pseudopeptides [pseudopeptides are peptides including one or more exotic aminoacid(s) in their sequence] with good MCH_1_ antagonism and fair *in vivo* stability. We succeeded in describing S38151 (Audinot et al., 2009), a potent peptide at MCH_1_ with antagonistic activity in the nanomolar range. The aim of the present study was to evaluate the possible inhibition of S38151 on food and water intakes, body weight, and motility after one-time or repeated daily peripheral administration in different rodent models of obesity. Confirmation of the MCH_1_-mediated actions of S38151 was tested in mice with genetic ablation of the receptor. Mice with genetic ablation of MCH_1_were used to confirm that the transient inhibition of S38151 onto the bodyweight was mediated by MCH_1_.

## Materials and methods

### Peptides

MCH and S38151 (Audinot et al., 2009) were obtained from Polypeptide Laboratories (Illkirch, France) and/or from Genepep (Saint Jean de Védas, France). They were of purity exceeding 97%. S38151 is a pseudopeptide, the sequence of which is: [p-guanidinobenzoyl-(Des-Gly10)-MCH (7–17)], or pGua-Cys-Met-Leu-Arg-Val-Tyr-Arg-Pro-Cys; both cysteines are linked together with a disulfide bridge. The molecular weight of S38151 is 1485 g/L. All of the various lots were systematically analyzed using mass spectrometry.

### S38151 stability studies

First, S38151 was incubated for 16 h at 4°C and 37°C in mouse plasma or in TRIS buffer (pH 7.4). Next, plasma proteins were precipitated with two volumes of acetonitrile. After centrifugation and dilution with a water/acetonitrile mixture (50/50 v/v), the supernatant was injected onto a triple quadripole mass spectrometer (Ultima, Micromass WATERS Corporation, Milford, USA). The liquid chromatographic system consisted of an Agilent 1100 analytic pump (Agilent, Santa Clara, USA) equipped with an Alpha Mos CTC autosampler (Alpha Mos, Toulouse, France). Mass detection was performed using the m/z transition 743.5 > 163, in positive electrospray. A second stability study was performed at a lower concentration (1 μM) and for 4 h, with the following sampling times: 0, 15, 45, 90, and 240 min. At each sampling time, 50 μL of plasma were precipitated with two volumes of acetonitrile, and after centrifugation were directly injected onto the LC-MS/MS system. The same treatment was used for the buffer samples. Although brains from S38151-treated animals were analyzed to determine the amount of compound detectable in the brain, none was detected (data not shown).

### Animals

These experiments were conducted with male Wistar and Zucker *fa/fa* rats (10–13 weeks of age, weighing 325–350 g) together with C57BL/6J mice, *ob/ob* mice, and MCH_1_-KO mice (14 weeks of age, weighing 25, 45, and 25 g, respectively) (Iffa Credo and Charles River, L'Arbresle, France). The animals were housed individually in a room with a 12-h light/dark cycle (lights on at 07:30 and off at 19:30), at 22 ± 3°C and 55% relative humidity. Normal food and tap water were available *ad libitum* unless otherwise stated. All animal procedures described in this study comply with French laws regulating animal experimentation (Decree No 87-848 19th October 1987 and the ministerial Decree of 10 April 1988) and were approved by the animal ethics committee of the Servier Research Institute.

### Intra-cerebroventricular cannula implantation

#### Acute i.c.v. injections in rats

Wistar and Zucker *fa/fa* rats were anesthetized with Forène (Abbott Laboratories, Queenborough, UK), and a stainless steel guide cannula (Plastic Products Co, Roanoke, Va, USA) was stereotaxically implanted into the right lateral ventricle at the following coordinates relative to the bregma: *AP* −0.8 mm, *L* = −1.2 mm, and *V* = −3.5 mm. After a 7-day recovery period, during which the animals were handled each day to minimize non-specific stress, they were lightly anesthetized with Forène. Human/mouse/rat MCH (1 μg in a volume of 2.5 μ L, Bachem, Voisins-le-Bretonneux, France) or an equivalent volume of artificial cerebrospinal fluid (CSF) was then injected through the intraventricular cannula. Immediately after injection, the animals quickly recovered and were returned to their home cages. Only those animals responding within 2 h with a robust increase in food intake, indicating correct cannula placement, were used in the experiments described below. Two days after MCH injection, the rats were randomly assigned to different groups and studied in one of the following protocols.

#### Acute central peptide administration and feeding studies in Wistar rats

Wistar rats were habituated for at least 2 weeks before experiment to a diet of food pellets (6 mm diameter) of the following composition: 67.5% food flour, 26.5% sucrose, 5% gum tragacanth, and 1.25% magnesium stearate (A03 UAR, Orge, France). The food pellets were present in a metal food hopper attached to the inside of each cage. At 09:00 on the day of study, the animals were lightly anesthetized with Forene® (Abbott, France). Thereafter, rats were injected intra-cerebroventricular (i.c.v.) with vehicle (artificial CSF) or with S38151 [0.5, 5, 10, 20, 30, or 50 nmol/kg (0.7, 1.4, 14, 28, 42, or 70 μg/kg, respectively)] in a volume of 2.5 μL, 20 min prior to the injection of human/rat NEI-MCH [5 nmol/kg (18.7 μg/kg) in a volume of 2.5 μL].

Food intake was measured at different times after peptide injection. Food spillage was carefully quantified during each time period, and intake measurements were corrected for this loss. At the end of the experiments, the animals were euthanized and the positions of the cannulae were assessed by the injection of 100 μ L Evans blue dye (2 mg/mL) followed by the visual examination of brain slices. Only data obtained from animals with correctly positioned cannulae were included in the final data analysis.

#### Daily central peptide administration and feeding studies in Zucker rats

The TSE Drinking and Feeding Monitor system was used to analyze ingestive behavior in these experiments (TSE Technical & Scientific Equipment GmbH, Bad Homburg, Germany). The rats' motility was also determined in these experiments using the MoTil® system (TSE). Zucker *fa/fa* rats were placed individually into plastic cages to which were attached both a feeding and a drinking sensor. The animals were habituated to normal food (A03 UAR, Orge, France) and water for at least 2 weeks before experimentation. One hour prior to the beginning of the dark phase, Zucker *fa/fa* rats were injected i.c.v. with different doses of S38151 [5, 10, or 20 nmol/kg (3.5, 14, or 28 μg/kg, respectively)] or with an equivalent volume of artificial CSF vehicle (10 μL). The injections were repeated daily for a total of 7 days. After 7 days, the injections were stopped and each measured parameter was followed during a 5-day washout period.

### Acute peripheral peptide administration and feeding studies in Zucker rats

Zucker fa/fa rats were habituated in the same conditions as Wistar rats (described in section “Acute Central Peptide Administration and Feeding Studies in Wistar Rats”). At 09:00 on the day of study, after fasting for 24 h, either vehicle (normal saline) or S38151 [30 mg/kg (20 μmol/kg)] was administered intraperitoneally (i.p.).

### Conditioned taste aversion

These experiments were performed according to previously described methods (Criscione et al., 1998; Welzl et al., 2001). In brief, Wistar rats were individually housed in plastic cages with free access to normal food and water. During a 7-day habituation period, the water bottle was removed from the cages overnight (from 16:00 to 10:00). At 10:00, water was made available to the animals from 250-mL plastic bottles to which a normal drinking spout was attached. The bottles were weighed before and after presentation to the animals to determine the quantity of water ingested during this period. After 4 days of this treatment regime, which served to habituate the animals to the experimental protocol and to randomize the animals, 0.2% saccharine was presented instead of water. Twenty minutes after the test period, the animals were injected i.p. with lithium chloride (75 mg/kg) or i.c.v. with S38151 [20 nmol/kg (28 μg/kg)] or vehicle (artificial CSF). Four, 7, 11, and 14 days later, the consumption of saccharine was determined. Considerable care was taken in these experiments to first prevent and then quantify any leakage of saccharine solution during the presentations.

### Pica

These experiments were performed with modifications to previously described methods (Madden et al., 1999). Rats were individually housed in wire-bottomed plastic cages with free access to both food pellets (6 mm diameter A03 UAR) and kaolin powder. The food pellets were presented in a small porcelain bowl on the floor of the cage. The kaolin powder was presented in a metal feeding dish attached to the inside of the cage. After a 2-week habituation period to these conditions, the animals were randomized into five treatment groups based on the quantity of food eaten during the stabilization period. In addition, those rats that consistently spilled the kaolin powder were eliminated from the study. One day after randomization, the animals were injected just before the beginning of the dark phase with either cyclophosphamide (100 mg/kg, i.p.), or S38151 [20 nmol/kg (28 μg/kg) i.c.v.] or vehicle [(artificial CSF) i.c.v.]. After injection, the quantity of kaolin and food ingested during the following 24-h period was determined. Food or kaolin spilled by the animals was collected at the end of the experiment, and total food and kaolin intake measurements were corrected for this loss.

### Construction of MCHr1^−/−^ mice

#### Construction of the targeting vector

**Figure 1:**
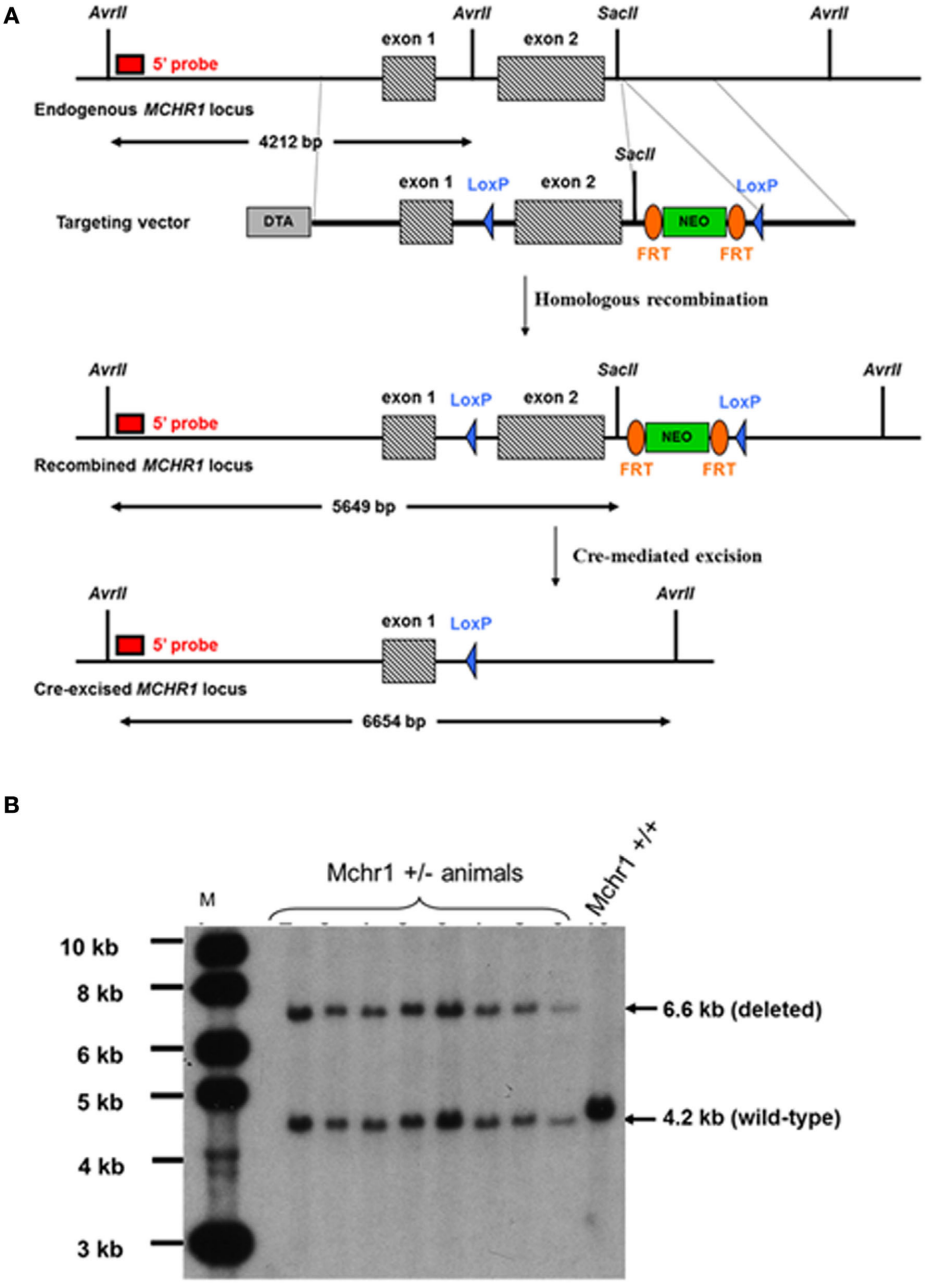
**Construction of MCHR1^−/−^ mice. (A)** Strategy for the construction of MCHR1^−/−^ mice. The *Mchr1* locus (containing exons 1–2) and the targeting construct [containing the neomycin (PGK-Neo) cassette with flanking segments homologous to the locus] are shown. The probe used in all Southern blot analyses was a 0.65-kb fragment located in a region upstream of exon 1. **(B)** Southern blot analysis of the offspring of MchR1-deleted mice, compared with C57BL/6J mice. Digestion of tail DNA with AvrII/SacII resulted in the following diagnostic fragments: a wild-type band (4.2 kb), and a deleted allele band (6.6 kb).

The MCHR1^−/−^ mouse model was custom-constructed by genOway (Lyon, France). The construction of the targeting vector and knock-in strategy were designed and performed by genOway (Lyon, France). A genomic clone containing the murine Mchr1 locus was isolated from a C57BL/6J RPCI-24 BAC genomic library by using a probe corresponding to the murine Mchr1 exon 2: one BAC clone (544N15) containing the Mchr1 locus. The genomic organization of the targeted locus was determined by subcloning the XhoI-SpeI genomic fragment into the pZErO™-2 vector (Invitrogen, Carlsbad, California). The 7.4-kb XhoI-SpeI genomic insert was sequenced and the Mchr1 sequence was generated. The genomic clone (containing the entire gene) was used to construct the targeting vector. Briefly, a 5.1-kb XhoI/ApaI fragment comprising Mchr1 exons 1 and 2 and a 1.1-kb ApaI/BglII fragment located downstream of Mchr1 exon 2 were used to flank a NEO cassette (FRT-PGK promoter-Neo^R^ cDNA-FRT-LoxP) (Figure 1A); a negative (DTA) selection cassette was introduced at the 5' end of the long arm of homology.

#### Screening of Mchr1-Recombined ES cell clones

NotI-linearized targeting vector was transfected into 129SvPas ES cells (genOway, Lyon, France) according to genOway's modified electroporation procedures (i.e., 10^8^ ES cells in the presence of 100 μg of linearized plasmid, 800 V, 300 μF). Positive selection was started 48 h after electroporation, by adding 200 μg/mL of G418 (150 μg/mL of active component, Life Technologies, Inc.). A total of 333 resistant clones were isolated and amplified in 96-well plates, and duplicates were made of the 96-well plates. The set of plates containing ES cell clones amplified on gelatin was screened by PCR and further confirmed by Southern blotting.

5' PCR screening conditions were: GW676 primer specific for a region upstream of *Mchr1* exon 1 (5'-GATATCAATTCGGGACACATGG-3') and GW681 primer specific for the Neo^R^ selection cassette (5'-TCTCGGCAGGAGCAAGGTGAGATGACAG-3'). PCR conditions were 94°C/2 min, and 35 cycles of (94°C/30 s, 59°C/30 s, and 68°C/6 min) followed by 68°C/10 min, resulting in a 6509-bp mutated allele. 3' PCR screening conditions were: GW668 primer specific for the Neo® selection cassette (5'-ATCAGGACATAGCGTTGGCTAC-3') and GW669 primer to hybridize the region downstream of the *Mchr1* gene (5'-ATGAGAAGTGACCAGCAAGAGC-3'). PCR conditions were 94°C/2 min, and 35 cycles of (94°C/30 s, 61°C/30 s, and 68°C/2 min), followed by 68°C/10 min, resulting in a 1609-bp recombined allele. Both PCR reactions were performed using Long Expand High Fidelity polymerase (Roche®) and reaction buffer 3. PCR products were then digested with PmeI to confirm the integration of the distal *loxP* site within the recombined allele. Briefly, for Southern blot analysis, genomic DNA was digested with AvrII/SacII and then hybridized with a 0.65-kb probe; *Mchr1* ± clones gave rise to a 4.2-kb wild-type signal and 5.6-kb recombined signal.

#### In vitro Cre-mediated deletion of Mchr1-Recombined ES cell clones

Two recombined ES cell clones were used to perform Cre-mediated deletion of the floxed *Mchr1* region. Supercoiled Cre-expressing plasmid was transfected into *Mchr1*-recombined ES cells (genOway, Lyon, France) according to genOway standard procedures. No selection was applied. One hundred ES cell clones were isolated and amplified in 96-well plates, and duplicates were made of the 96-well plates. The set of plates containing ES cell clones amplified on gelatin was screened by PCR and further confirmed by Southern blotting. The PCR screening conditions were: GW787 primer specific for *Mchr1* exon 1 (5'-AGCTCTGAAGGAGAAGGGAATG-3') and GW669 primer to hybridize the region downstream of the *Mchr1* gene (5'-ATGAGAAGTGACCAGCAAGAGC-3'). The PCR conditions were 94°C/2 min, and 35 cycles of (94°C/30 s, 59°C/30 s, and 68°C/6 min), followed by 68°C/10 min, resulting in a 4481-bp recombined allele and a 1564-bp deleted allele. The PCR reaction was performed using Long Expand High Fidelity polymerase (Roche®) and reaction buffer 3. Briefly, for Southern blot analysis, genomic DNA was digested with AvrII/SacII and then hybridized with a 0.65-kb probe. The *Mchr1* wild-type allele gave rise to a 4.2-kb signal, the *Mchr1*-recombined allele gave rise to a 5.6-kb signal, and the *Mchr1* deleted allele gave rise to a 6.6-kb signal.

#### Generation of chimeric mice and breeding scheme

One floxed mutated *Mchr1* ES cell clone (clone #2A9-2A10) was microinjected into C57BL/6J blastocysts, and gave rise to male chimeras with a significant ES cell contribution (as determined by Agouti coat color). After mating with C57BL/6J females, germline transmission was confirmed by the genotyping of tail DNA from offspring using PCR and Southern blot analysis. Knock-out heterozygous animals were screened as described in section “Screening of Mchr1-Recombined ES Cell Clones.” PCR screening conditions were: GW787 primer specific for *Mchr1* exon 1 (5'-AGCTCTGAAGGAGAAGGGAATG-3') and GW669 primer to hybridize the region downstream of the *Mchr1* gene (5'-ATGAGAAGTGACCAGCAAGAGC-3'). PCR conditions were 94°C/2 min, and 35 cycles of (94°C/30 s, 59°C/30 s, and 68°C/6 min), followed by 68°C/10 min, resulting in a 3.1-kb signal for the wild-type allele and 1.6-kb signal for the deleted allele. The PCR reaction was performed using Long Expand High Fidelity polymerase (Roche®) and reaction buffer 3. Briefly, for Southern blot analysis, genomic DNA was digested with AvrII/SacII and then hybridized with a 0.65-kb probe. The wild-type *Mchr1* allele gave rise to a 4.2-kb signal and the deleted *Mchr1* allele gave rise to a 6.6-kb signal (Figure 1B). F1 male and female heterozygous animals were interbred to obtain *Mchr1*-null mice, and offspring were also screened by PCR and Southern blot analysis as described in this section.

### Food intake in C57Bl/6J and ob/ob mice

For the MCH daily repeated i.c.v. injection studies, 13-week-old female C57BL/6J and *ob/ob* mice were used (Harlan, Gannat, France).

### Food intake induced obesity in C57BL/6J, ob/ob, and MCHr1-KO mice

In these studies, 13-week-old female C57BL/6J, ob/ob, and MCH_1_-KO mice were used. The mice were maintained on a 12-h light/dark cycle (lights on at 07:30 and off at 19:30) at 22 ± 3°C, and supplied with food pellets (6 mm diameter A03, UAR Laboratory chow, Epinay Villemuisson France) and tap water *ad libitum*. To induce obesity in C57BL/6J (creating diet-induced obese, or DIO, mice), mice were fed a high-fat diet (60 kcal% fat, 20 kcal% protein, 20 kcal% carbohydrate, ref D12492 from Research Diets, New Brunswick, NJ08901, USA) for 8 weeks starting at 4 weeks of age. In these experiments, DIO mice, ob/ob mice, and MCH^−/−^_1_ mice were individually housed in a modular chamber that was placed on an activity platform to concurrently measure the food intake, water intake, and activity of the mice (ADDENFI, Les Cordeliers, Paris, France). For at least 2 weeks before experimentation, mice were habituated to a diet of 6 mm diameter food pellets of the following composition: 67.5% food flour, 26.5% sucrose, 5% gum tragacanth, 1.25% magnesium stearate (A03 UAR, Orge, France), and to i.p. injection. After this habituation period, food intake, drink intake, and body weight were measured for 3 consecutive days. The mice were randomized into three groups based on their food and water intake over the three preceding 24-h periods, and on their body weight. Next, the quantity of food eaten over each 24-h period during the 5 days of i.p. treatment with S38151 [10 and 30 mg/kg/day (7 and 20 μmol/kg/day)] or vehicle was determined and corrected for spillage.

### Data and statistical analysis

Data were grouped together and presented as mean ± standard error (SEM). The individual statistical tests that were used in these studies are described in detail in the figure legends. In each case, *P*-values less than 0.05 were considered statistically significant.

## Results

### S38151 characteristics and serum stability

**Figure 2:**
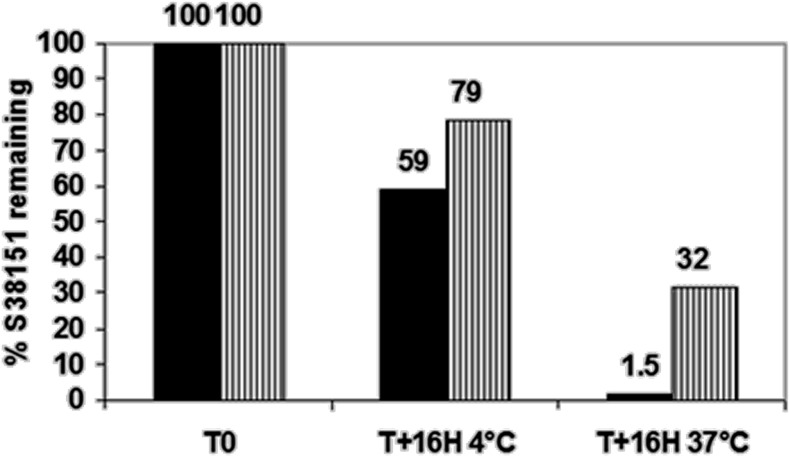
**Studies regarding the stability of S38151.** A solution of S38151 (40 μM) was incubated with either Tris buffer, pH 7.4 (hatched bars), or mouse plasma samples (dark bars) for 16 h at 4°C or 37°C. The samples were analyzed using liquid chromatography coupled to mass spectrometry. Experiments were repeated three times. The chart presented is representative of the experimental results.

S38151 was synthesized during a process that was attempting to find peptides or pseudopeptides with affinity at MCH_1_ receptor that also had greater stability in biological media. This pseudopeptide is derived from another, very similar agonist, pGua (MCH7-17) (Audinot et al., 2001a, 2009). In Chinese hamster ovary cells over-expressing MCH_1_, S38151 exhibited a K_i_ of 80 nM in a [^125^I]-S36057 binding assay and was a potent and full antagonist (*KB* = 4.3 nM in a GTPγS binding assay, and *KB* = 210 nM in a calcium flux assay) (Audinot et al., 2009). This activity was specific to MCH_1_, since it has no activity with respect to MCHR2 at concentrations of less than 10 μM. This compound was also evaluated on a series of 70 targets, comprising monoaminergic and peptidergic receptors as well as enzymes, ionic channels, and transporters. S38151 did not display any activity regarding these targets at concentrations of less than 10 μM (data not shown). The peptide was incubated either in a buffered solution (pH 7.4) or in mouse plasma for 16 h, at either 4°C or 37°C. Figure 2 clearly shows that at 37°C, a minimal portion of S38151 is detectable, suggesting that it has limited stability in plasma.

### The effect of a single i.c.v. injection of S38151 on NEI-MCH-induced food intake in wistar rats

**Figure 3:**
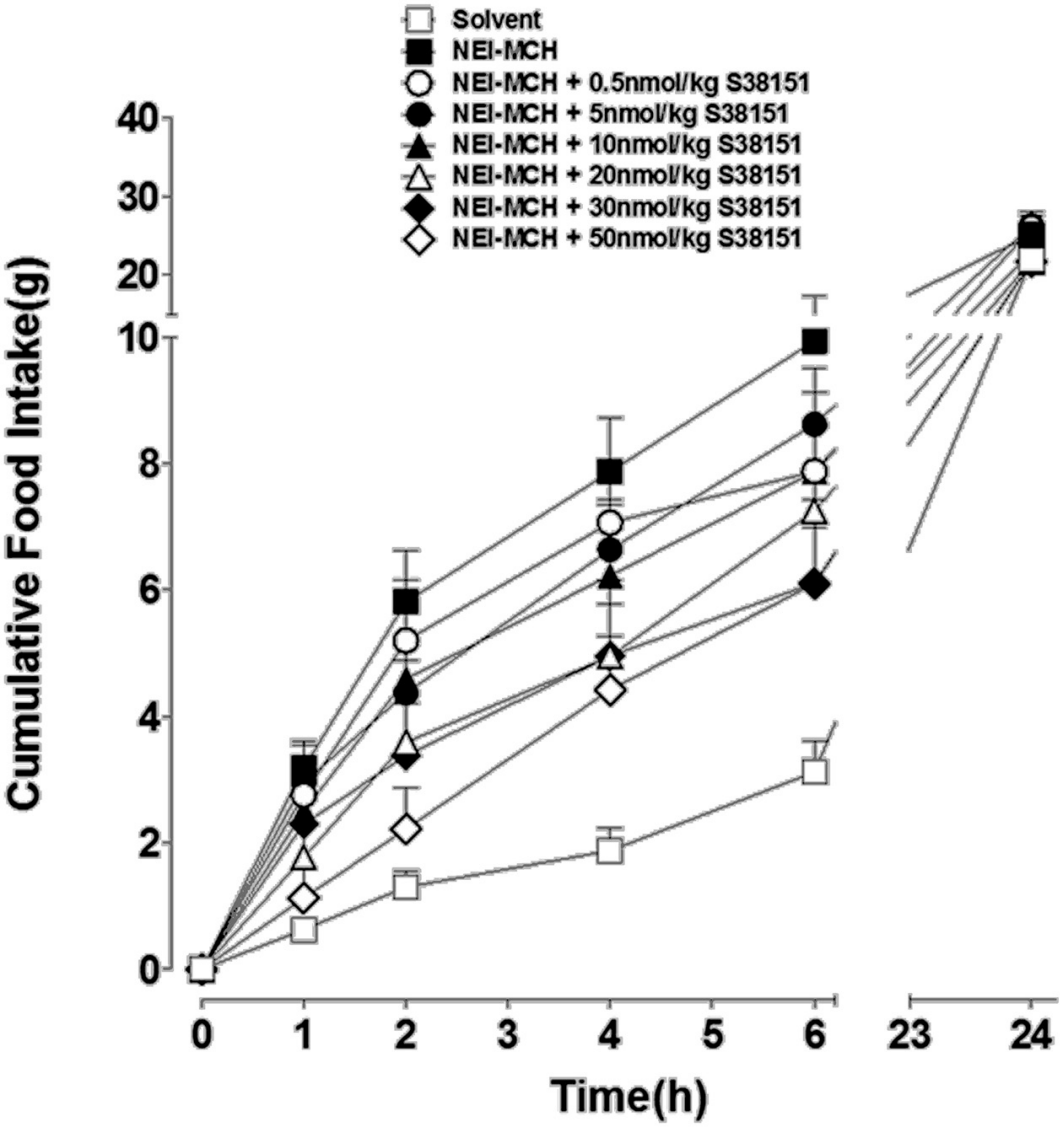
**The effect of S38151 on NEI-MCH-induced food intake in Wistar rats after acute central administration.** Satiated rats were injected (i.c.v.) at the beginning of the light phase with 5 μg NEI-MCH, 5 μg NEI-MCH + differing doses of S38151 (0.5–50 nmol/kg), or an equivalent volume of artificial cereblospinal fluid vehicle (5 μL). Cumulative food intake was measured at 1, 2, 4, 6, and 24 h post-injection. Results are expressed as mean ± SEM (*n* = 10–11 rats per group). Repeated measures Two-Way ANOVA (treatment × time) was followed by *post hoc* Student-Neuman-Keuls analysis to compare all groups of animals.

The acute effects of a single i.c.v. injection of S38151 on NEI-MCH-induced food intake in Wistar rats are summarized in Figure 3. During the first 2 h after its injection, NEI-MCH produced a significant increase in food intake above the corresponding values observed in vehicle-injected control animals (T1h: 3.18 g ± 1.09 vs. 0.45 g ± 0.19; T2h: 2.73 g ± 0.89 vs. 1.25 g ± 0.38). Relative to control values, the increase in food intake induced by NEI-MCH became progressively greater during the following 4-h period (from T1 h to T4 h). During the first and second hours, the MCHR1 antagonist S38151 produced a statistically significant dose-related inhibition of the increase in food intake produced by NEI-MCH. Nearly complete inhibition of NEI-MCH-induced food intake was observed at the dose of 50 nmol/kg S38151. Thereafter, for each dose of S38151, food intake increased essentially in parallel with the food increase in the NEI-MCH-treated group. Food intake during the entire 24-h period in the control group was not significantly altered by NEI-MCH, or by any combination of NEI-MCH and S38151.

### The effects of daily S38151 i.c.v. injections on food intake, body weight, and motility in Zucker fa/fa rats

**Figure 4:**
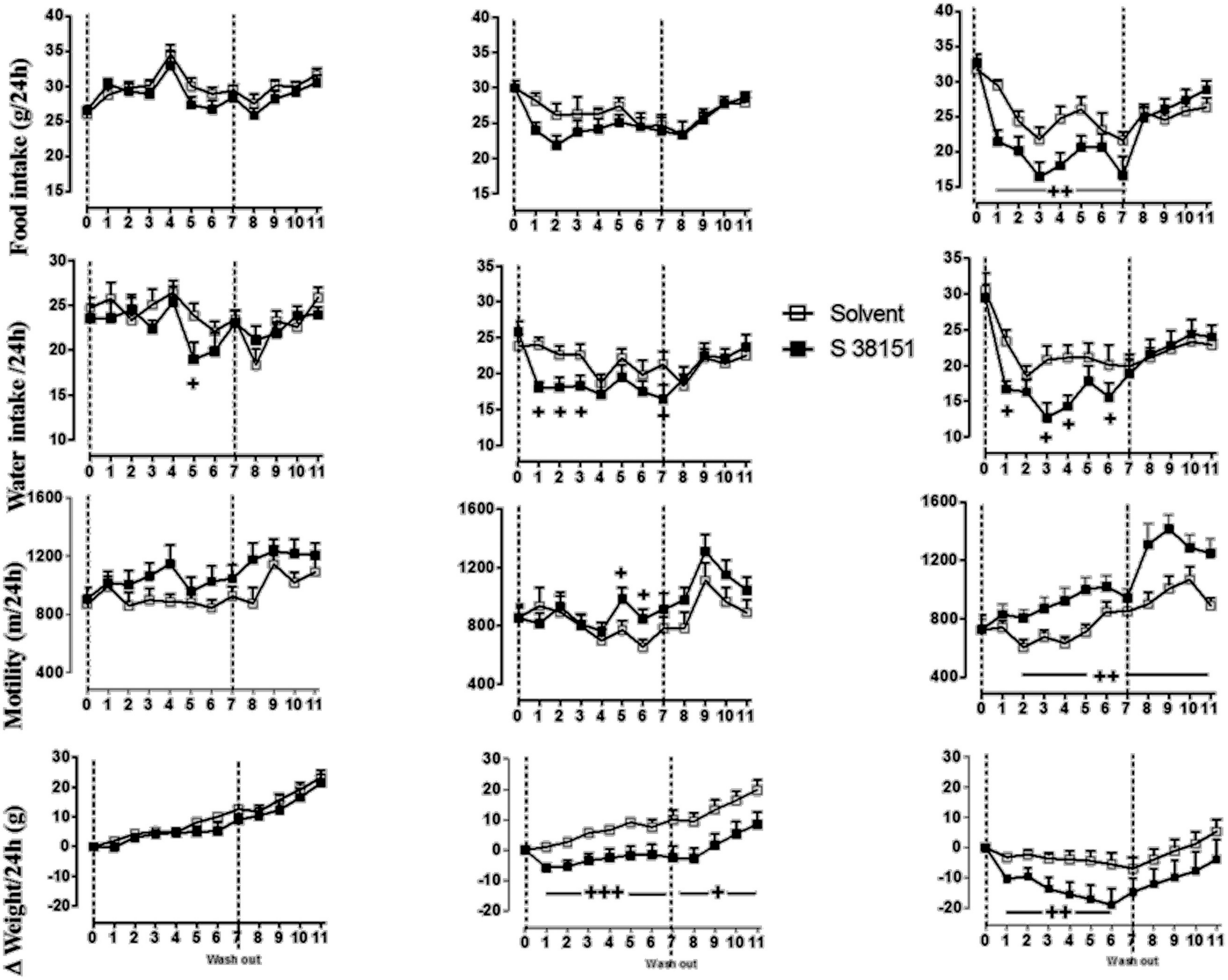
**The effects of repeated daily central administration of S38151 on food intake, water intake, body weight, and motility in Zucker *fa/fa* rats.** One hour prior the beginning of the dark phase, different doses of S38151 (5, 10, or 20 nmol/kg) or vehicle were injected (i.c.v.) once daily for 7 days, followed by a washout period of 5 days. Results are expressed as mean ± SEM (*n* = 8 rats per group). Repeated measures Two-Way ANOVA (treatment × time) was performed with a repeated measure of the factor “time.” Significant treatment and interaction effects are shown on the graph. ^+^*P* < 0.05; ^++^*P* < 0.01; ^+++^*P* < 0.001.

The effects of daily S38151 i.c.v. injection on the measured parameters are depicted in Figure 4. During the 7-day experimental period, injection of 28 μg/kg (5 nmol/rat) S38151 1 h before the dark phase resulted in dose-dependent inhibition of food intake. However, the change in food intake only reached statistical significance at the dose of 20 nmol/kg (28 μg/kg) S38151. Immediately after the injections were terminated, food intake returned to control levels for the duration of the washout period.

In contrast to food intake, water consumption appeared to be more sensitive to inhibition of MCHR1; water intake was significantly inhibited in response to all injected doses of S38151 except for the 5 nmol/rat dose. Immediately after the injections were terminated, water intake returned to control levels during the washout period. In a manner similar to water intake, motility also appeared to be more sensitive to the effects of MCH_1_ blockade; motility was significantly increased at both the 10 nmol/kg and 20 nmol/kg doses of S38151. Notably, the increase in motility was not terminated after the S38151 injections were stopped. This finding was most marked during the washout period after administration of the 20 nmol/kg dose of S38151, during which motility remained significantly elevated. During the 7-day experimental period, S38151 produced a dose-dependent reduction in body weight gain compared with control rats at the 10 and 20 nmol/kg doses. After the injections were stopped, body weight gain exhibited a tendency to return to control values. However, body weight gain remained significantly less than control values during the washout period after injection of 10 nmol/kg S38151.

### The effects of S38151 on conditioned taste aversion and pica in Zucker fa/fa rats

**Figure 5:**
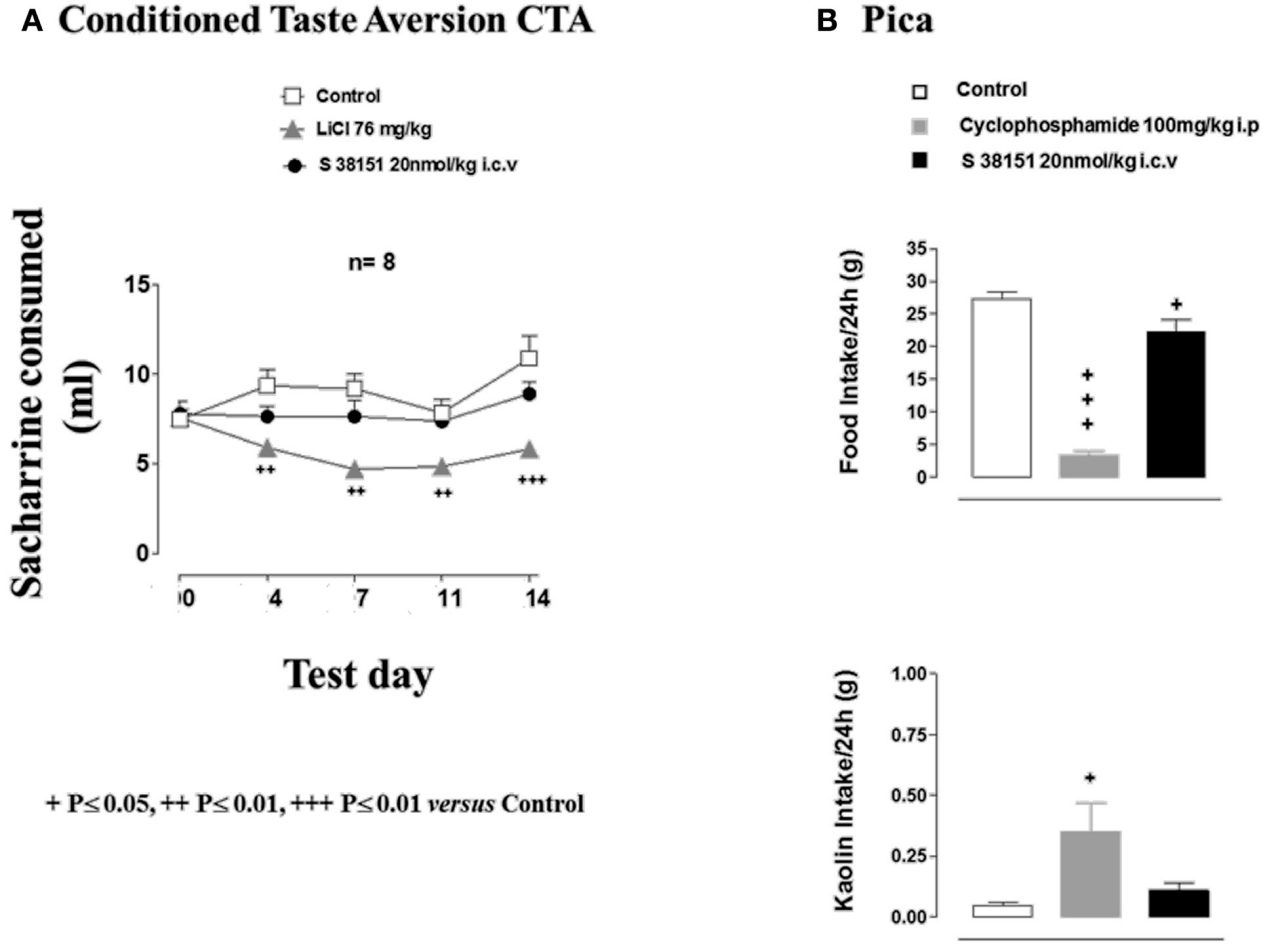
**The effects of S38151 on conditioned taste aversion and pica in Zucker *fa/fa* rats. (A)** In the conditioned taste aversion test, after a period of habituation of the animals to the experimental protocol, Zucker *fa/fa* rats were presented with 0.2% saccharine for 20 min after drinking water was removed from their cages for 18 h (test day 0). Animals were then injected (i.p.) with vehicle or lithium chloride, or with S38151 (20 nmol/kg, i.c.v). On test days 4, 7, 11, and 14, rats were presented with 0.2% saccharine, and the quantity consumed during a 20-min period was determined. Results are expressed as mean ± SEM (*n* = 10–11 rats per group). Repeated measures Two-Way ANOVA (treatment × time) was followed by *post hoc* Student-Neuman-Keuls analysis to compare all groups of animals. ^++^*P* < 0.01, ^+++^*P* < 0.001. **(B)** In the pica test, Zucker *fa/fa* rats were injected (i.c.v.) with 20 nmol/kg S38151, or an equivalent volume of artificial cerebrospinal fluid vehicle. Food and kaolin intake during the following 24-h period were each measured. Results are expressed as mean ± SEM (*n* = 8 rats per group). One-Way ANOVA was followed by Dunnett's test, which compared the treated group with the control group. ^+^*P* < 0.05, ^+++^*P* < 0.001.

The results of the conditioned taste aversion test are shown in Figure 5A. The consumption of 0.2% saccharine during the first exposure (test day 0) before treatment was well matched between the three groups. Subsequent injection of lithium chloride resulted in a significant reduction in 0.2% saccharine consumption during test days 4, 7, 11, and 14 compared with the control group. In contrast, S38151 injection did not affect 0.2% saccharine consumption.

In the Pica test, vehicle-injected control animals ate 27 ± 1 g of normal food and very little kaolin (0.05 ± 0.01 g) during the 24-h experimental period (Figure 5B). Cyclophosphamide (i.p. injection) resulted in the nearly complete suppression of spontaneous food intake, which was accompanied by a significant increase in kaolin intake. In contrast, i.c.v. injection of S38151 [20 nmol/kg (28 μg/kg)] significantly reduced food intake but did not significantly alter kaolin intake compared with control values.

### The effect of a single i.p. injection of S38151 on food intake in fasted Zucker fa/fa rats

**Figure 6:**
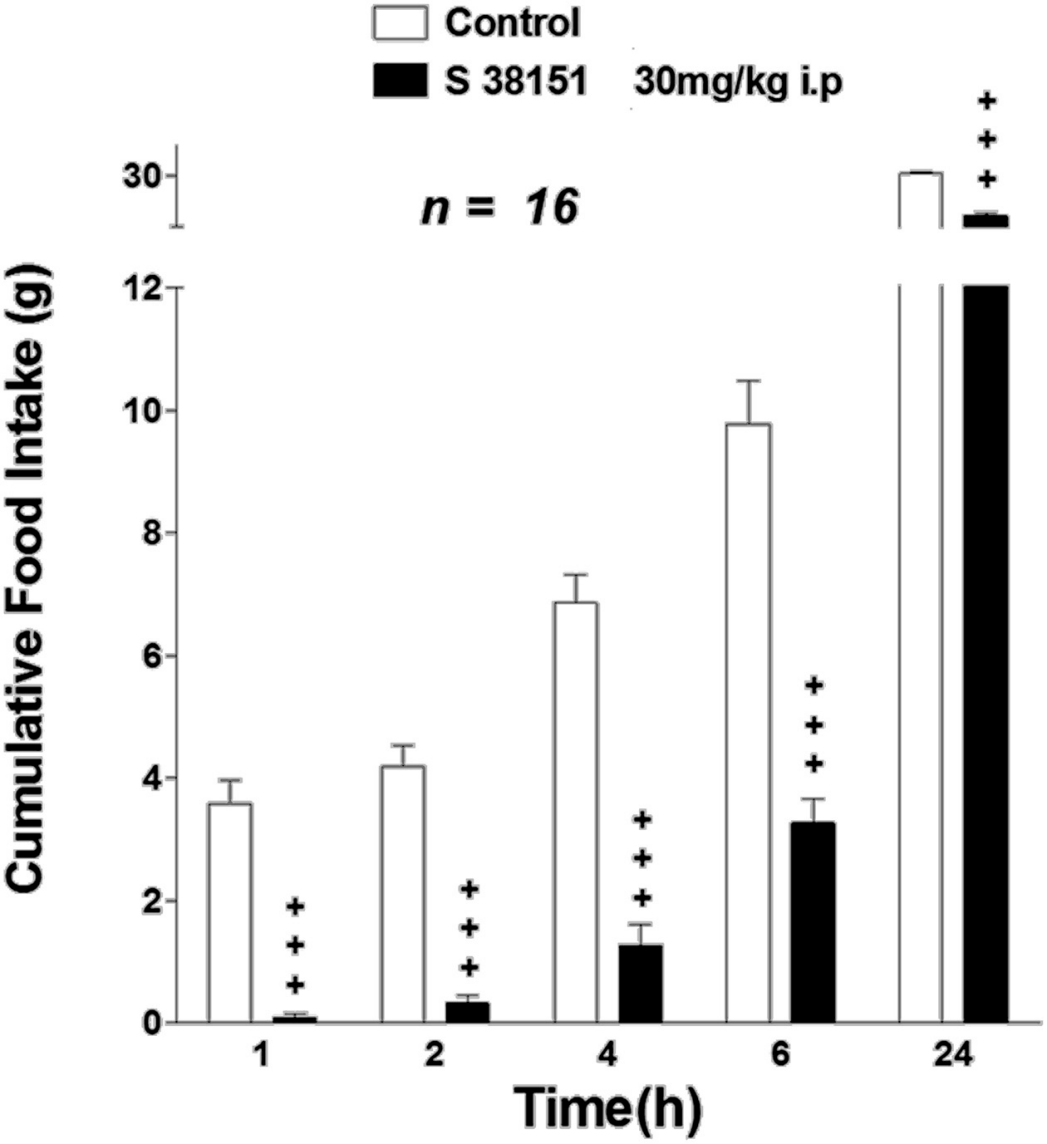
**The effect of S38151 on food intake in Zucker *fa/fa* rats after acute peripheral administration.** Rats were fasted for 24 h, and then injected (i.p.) at the beginning of the light phase with vehicle (normal saline) or 30 mg/kg S38151. Cumulative food intake was measured 1, 2, 4, 6, and 24 h after injection. Results are expressed as mean ± SEM (*n* = 16 rats per group). Repeated measures Two-Way ANOVA (treatment × time) was followed by *post hoc* Student-Neuman-Keuls analysis to compare all groups of animals. ^+^*P* < 0.05.

The acute effect of i.p.-injected S38151 on food intake in Zucker fa/fa rats is summarized in Figure 6. After a 24-h period of food restriction, rats ate 3.6 ± 0.4 g during the first hour after the food pellets were put back. S38151 (i.p. injection) at the dose of 30 mg/kg (approximately 60 nmol/rat) 1 h before re-feeding induced a dramatic fall in food intake from the first hour (0.09 ± 0.06 g) to 6 h compared with the control group. This decrease maintained statistical significance up to 24 h after drug administration.

### The effects of daily i.c.v. injection of MCH on food intake and body weight in ob/ob mice

#### Food intake

**Figure 7:**
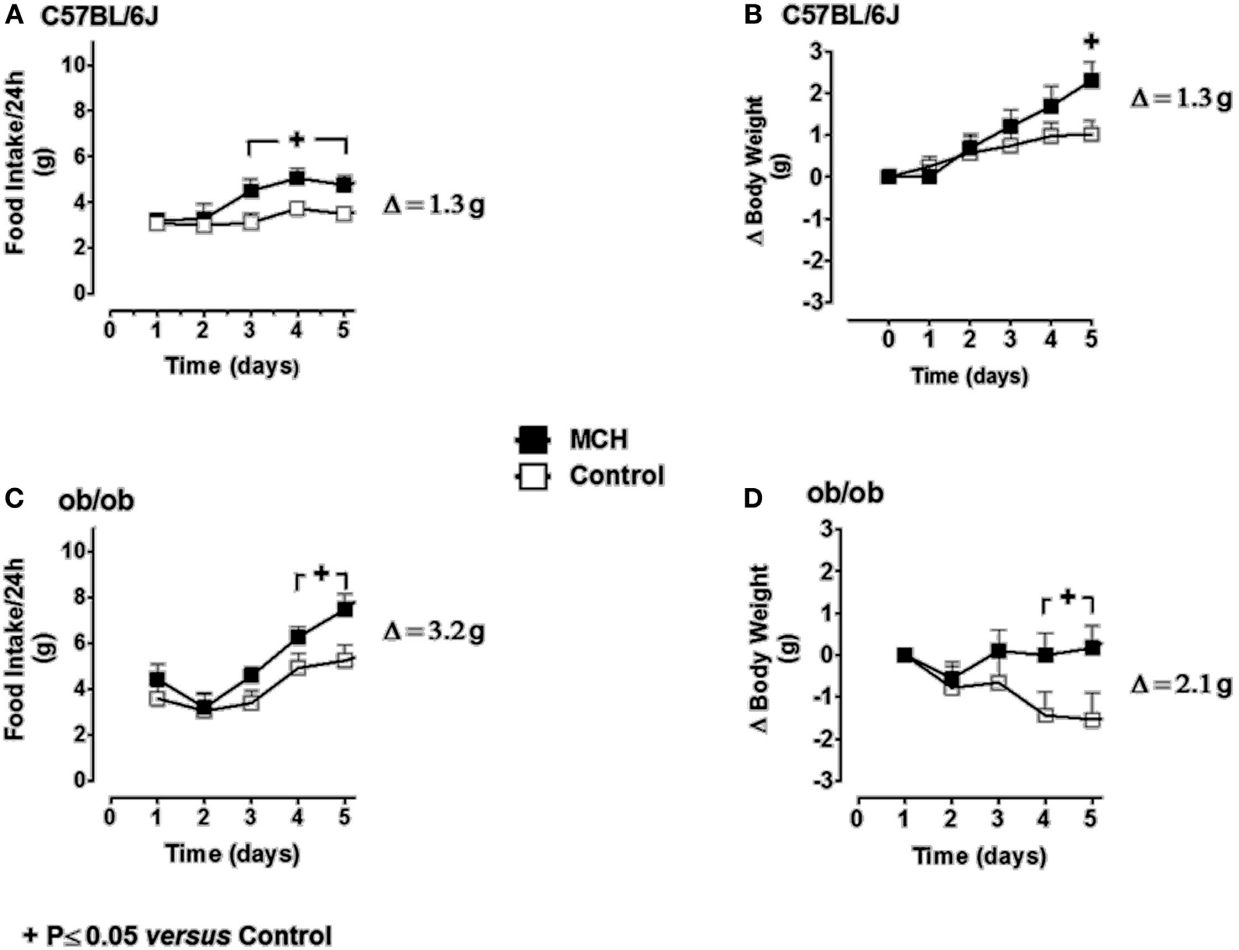
**The effect of repeated daily central administration of MCH to lean (C57BL/6J) and obese (*ob/ob*) mice.** C57BL/6J (**A** and **B**) and *ob/ob* (**C** and **D**) mice were administered MCH (5 μg/mouse/day) or artificial cerebrospinal fluid vehicle through i.c.v. injections for 5 consecutive days. Results are expressed as mean ± SEM (*n* = 10 mice per group). Repeated measures Two-Way ANOVA (treatment × time) was followed by complementary analysis of the treatment's effect at a fixed level of time to compare all groups of mice with the control group. ^+^*P* < 0.05.

Food intake in vehicle-infused C57BL/6J mice ranged from 3 to 4 g/day during the 5-day experimental period (Figure 7A). Infusion of MCH at 5 μg/mouse/day (100 nmol/kg/day) resulted in a sustained and significant increase in food intake above the values observed in vehicle-infused mice on day 5 (Δ = +1.3 g vs. controls). Food intake in ob/ob mice (Figure 7C) ranged from 3.5 to 5 g/day during the experimental period, and was significantly greater than that observed in C57BL/6J mice. Infusion of MCH at 5 μg/mouse/day in ob/ob mice significantly increased food intake above the values obtained in vehicle-infused control animals. The increase in food intake produced by MCH infusion in ob/ob mice on day 5 was significantly greater than that observed in lean mice (Δ = +3.2 g vs. controls).

#### Body weight

In C57BL/6J mice, body weight remained stable during the 5-day experimental period (Figure 7B). Infusion of MCH resulted in a significant increase in body weight on day 5 (Δ = +1.3 g vs. controls). Body weight tended to decrease slightly during the experimental period in vehicle-infused ob/ob mice (Figure 7D). However, infusion of MCH reversed this trend, and body weight had increased significantly above control levels after 5 days of treatment (Δ = +2.1 g vs. controls). The increase in body weight in MCH-infused ob/ob mice on day 5 was significantly greater than that observed in either group of lean mice.

### The effects of daily S38151 i.p. injection on food and water intake, body weight, and motility in ob/ob mice

**Figure 8:**
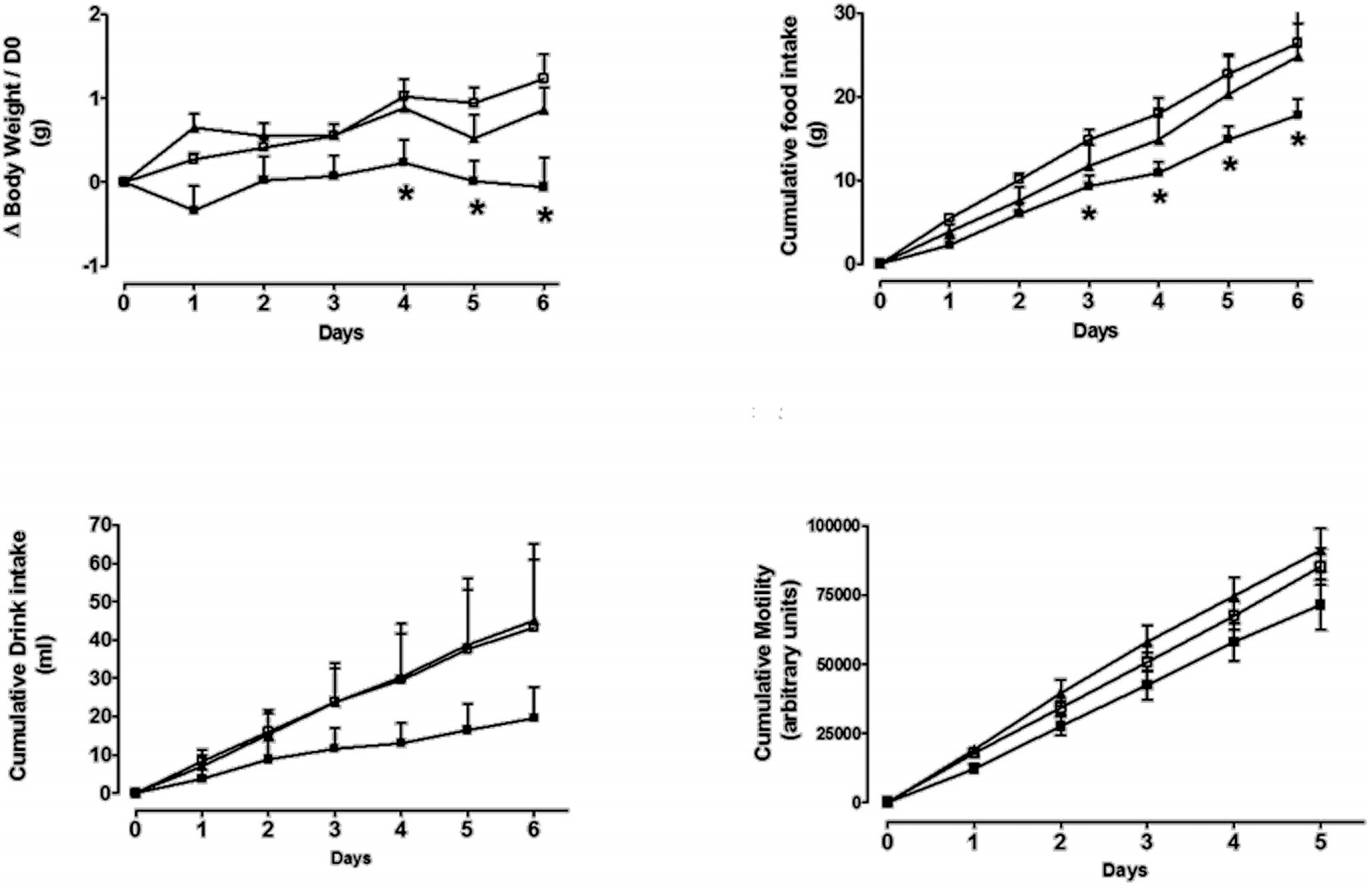
**The effects of repeated daily S38151 injection on food and water intake, body weight, and motility in *ob/ob* mice.** Genetically modified *ob/ob* mice were administered S38151 (10 and 30 mg/kg, i.p.) or vehicle (normal saline i.p.) for 6 consecutive days. Results are expressed as mean ± SEM (*n* = 8 mice per group). White squares for controls, dark squares for 10 mg/kg, and dark triangles for 30 mg/kg. Repeated measures Two-Way ANOVA (treatment × time) was followed by complementary analysis of each treatment's effect at a fixed level of time to compare all groups of mice with the control group. ^*^P < 0.05.

The effects of daily i.p. injection of S38151 on the measured parameters are depicted in Figure 8. During the 6-day experimental period, i.p. injection of S38151 1 h before the dark phase produced a dose-dependent inhibition of food intake that reached significance starting on day 3 only at the highest dose of 30 mg/kg (20 μmol/kg/day). Water intake presented the same profile, but did not reach statistical significance because of the greater variability in water consumption among mice. During the 6-day experimental period, S38151 produced a dose-dependent reduction in body weight compared with control mice, with the 30 mg/kg dose reaching statistical significance at days 1, 4, 5, and 6. In contrast, motility appeared to be similar among all groups. On day 6, an improvement of metabolic parameters (significant reductions in glycemia and insulinemia) was observed at a dose of 30 mg/kg (data not shown).

### The effects of daily i.p. injection of S38151 on food intake, body weight, and motility in DIO mice

**Figure 9:**
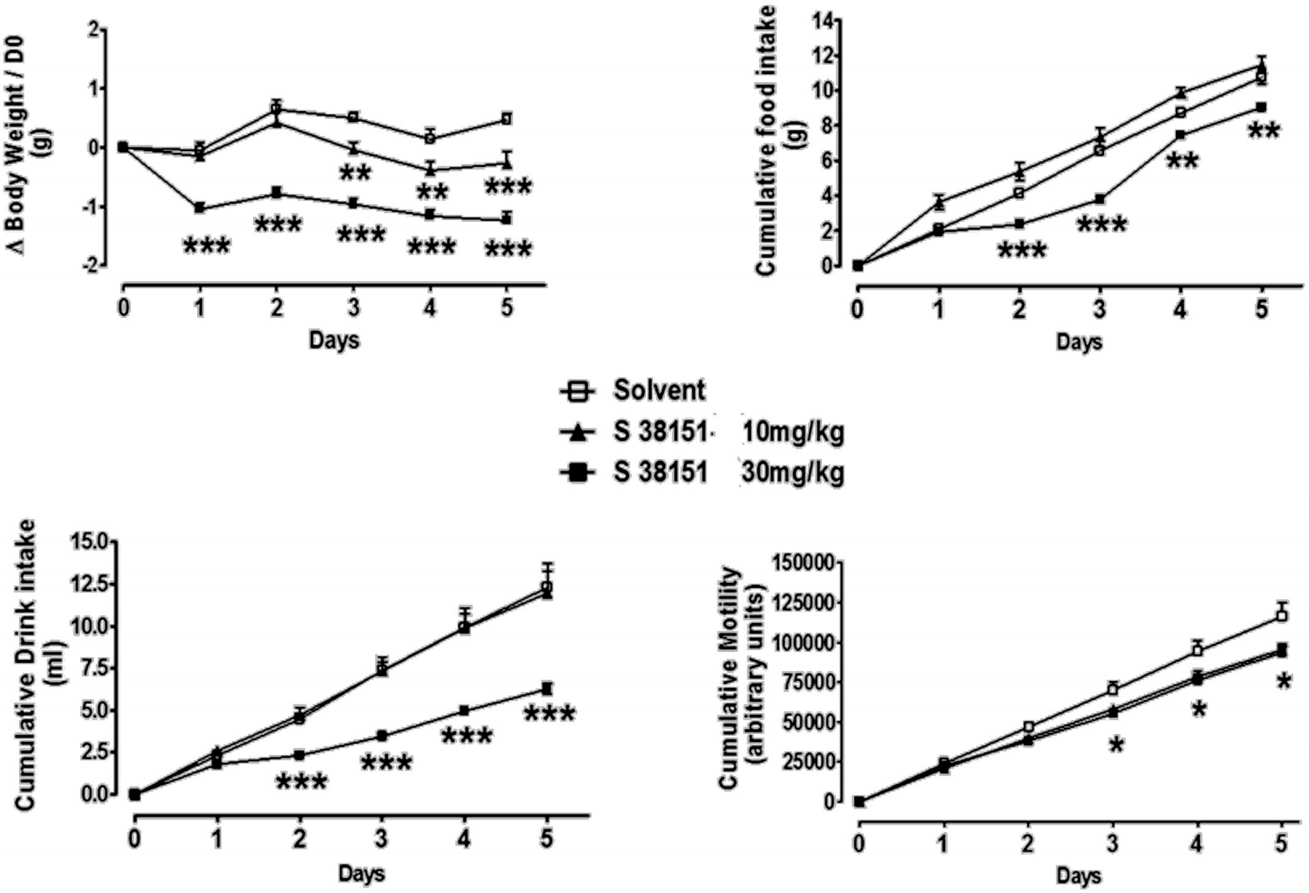
**The effects of repeated daily S38151 injection on food and water intake, body weight, and motility in diet-induced obese (DIO) mice.** DIO mice were administered S38151 (10 and 30 mg/kg, i.p.) or vehicle (normal saline i.p.) for 5 consecutive days. Results are expressed as mean ± SEM (*n* = 8 mice per group). Repeated measures Two-Way ANOVA (treatment × time) was followed by complementary analysis of each treatment's effect at a fixed level of time to compare all groups of mice with the control group. ^*^P < 0.05, ^**^P < 0.01, ^***^P < 0.001.

The effects of daily i.p. injection of S38151 on the measured parameters are depicted in Figure 9. During the 5-day experimental period, i.p. injection of S38151 1 h before the dark phase produced a dose-dependent inhibition of food and drink intake that reached significance beginning on day 2 only at the 30 mg/kg dose. A reduction in body weight gain was observed at the 10 and 30 mg/kg doses (7 and 20 μmol/kg/day, respectively), which reached statistical significance at day 3 and day 1, respectively, compared with control mice. S38151 induced a similar significant reduction in motility at the two tested doses from day 3 to day 5. At day 6, an improvement of metabolic parameters (significant reductions in glycemia and insulinemia) was observed at a dose of 30 mg/kg (data not shown). In another experiment, daily i.p. administration of 30 mg/kg S38151 for 16 days induced a significant reduction of body weight gain that was accompanied by reduced glycemia and insulinemia, as well as a significant reduction of triglycerides, free fatty acids, and fat mass (data not shown).

### The effects of daily i.p. injection of S38151 on food intake, body weight, and motility in MCH_1_-KO mice

**Figure 10:**
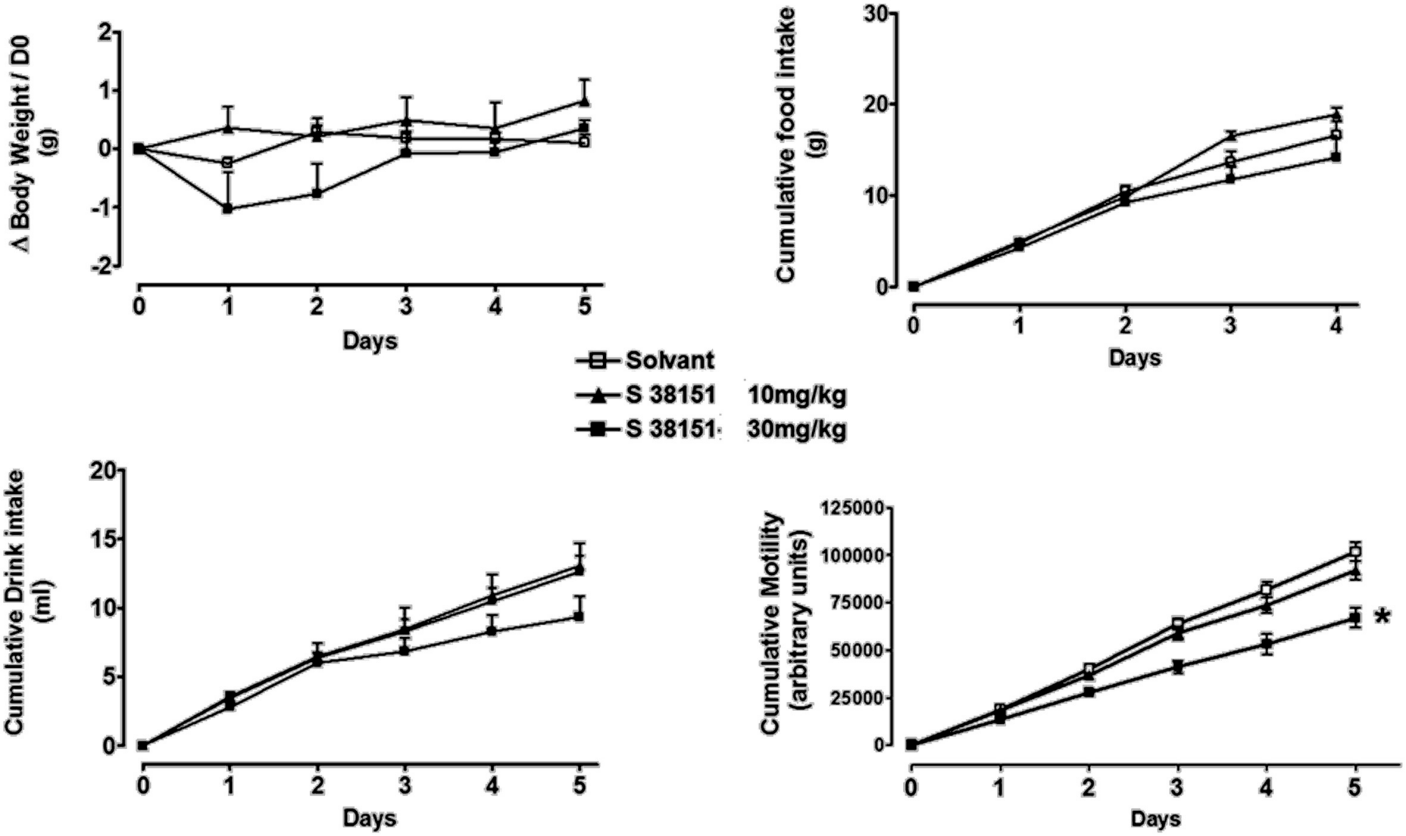
**The effects of repeated daily S38151 injection on food and water intake, body weight, and motility in MCHR1-KO mice.** MCH_1_-KO mice were administered S38151 (10 and 30 mg/kg, i.p.) or vehicle (normal saline i.p.) for 5 consecutive days. Results are expressed as mean ± SEM (*n* = 8 mice per group). Repeated measures Two-Way ANOVA (treatment × time) was followed by complementary analysis of each treatment's effect at a fixed level of time to compare all groups of mice with the control group. ^*^P < 0.05.

During the 5-day experimental period, i.p. injection of S38151 1 h before the dark phase did not induce any significant effects on the food intake, drink intake, or body weight of MHCR1-KO mice; however, a significant reduction in motility was observed at the highest dose of 30 mg/kg (20 μmol/kg/day) (Figure 10).

## Discussion

In the present study, we have investigated the effects of a selective MCH_1_ peptide antagonist, S38151 (Audinot et al., 2001a, 2009), on food intake, drinking, body weight, and/or motility in lean rats and mice, as well as in Zucker *fa/fa* rats, *ob/ob* mice, and DIO mice. Central acute administration of S38151 inhibited proMCH131-165 (MCH-NEI peptide)-induced feeding in lean animals in a dose-dependent manner. This effect lasted for over 6 h, in agreement with the estimated concentration of compound still present in plasma. In several obese rodent models (7-day treatment in Zucker *fa/fa* rats, 6-day treatment in *ob/ob* mice, and 5-day treatment in DIO mice), S38151 produced a robust and dose-dependent reduction in food intake and body weight gain. Drinking behavior was also inhibited in Zucker *fa/fa* rats and DIO mice. However, the cessation of S38151 injections resulted in a rapid resumption of feeding and drinking behaviors in Zucker *fa/fa* rats, a transitory action that greatly contrasted with the persistent weight loss. Repeated daily blockade of MCH_1_ receptors with S38151 reduced food intake and body weight with attenuated magnitude in leptin-deficient *ob/ob* mice relative to that observed in DIO mice. This observation was also reported with another MCH_1_ antagonist (Kowalski et al., 2006), presumably owing to the emergence of multiple alterations in leptin-responsive neuronal pathways implicated in the feeding behavior of *ob/ob* mice.

To clearly establish the involvement of MCH_1_ in the mode of action of S38151 on feeding and weight controls, its effects were evaluated in MCH_1_-KO mice. Consistent with direct MCH_1_ antagonism, S38151 did not produce any significant effect on feeding, drinking, or body weight gain in MCH_1_-KO mice treated for 5 days. Combined with the growing literature on MCH_1_ antagonists (see reviews Mendez-Andino and Wos, 2007; Johansson, 2011), these data strengthen the validation of MCH_1_ as a strong candidate for developing anti-obesity drugs.

Intriguingly, repeated daily MCH_1_ antagonism by S38151 induced persistent hypermobility in Zucker *fa/fa* rats, but did not affect locomotor activity in *ob/ob* mice, and even reduced motility in DIO mice. The differential response may result from differences in species (rat vs. mouse) or genetic background (*ob/ob* vs. C57BL/6J), or may be related to an off-target mechanism. When treated with S38151 for 5 days, MCH_1_-KO mice exhibited a reduction in motility similar to that noted in DIO mice. Therefore, the observed disparity in motor activity likely arose from off-target (i.e., MCH_1_-independent) effects of S38151.

In recent years, MCH_1_ antagonists have been described as central to the regulation of energy expenditure and food intake. However, unlike for neuropeptide Y receptor 5 antagonists [see Block et al. (2002) and references therein], data suggest that small molecule antagonists of MCH_1_ would have much broader activity, especially regarding mood regulation (and more generally, depression). Vast programs of drug synthesis and testing have been published in the literature. One of the surprising key features of those programs is that it appeared to be difficult to find compounds with potent antagonists against MCH_1_ that lacked activity regarding the hERG channel [see Mendez-Andino and Wos for a complete review (Mendez-Andino and Wos, 2007)].

Regarding the onset of obesity, it is well-known that the adipocyte has a central role, so it seemed interesting to find a way to act directly upon this cell type. MCH_1_ is expressed in adipocytes, suggesting that fat cells may be targets of MCH antagonists under physiological conditions (Bradley et al., 2000). This hypothesis was confirmed by our study using DIO mice treated with daily S38151 injections for 16 days, in which a reduction in fat mass was observed concomitant with improvements in glucose and lipid profiles. Indeed, acting through the central control of appetite has often failed, particularly for two reasons: (1) the number of “rescue” systems in the brain circuits is so important that inhibiting one pathway would lead to the “takeover” of one or several alternate pathways (Nair and Ren, 2009); (2) in some cases acting on a central system, such as the MCH-ergic system, to control appetite has promoted adverse effects, and particularly pro-depressive effects (Roy et al., 2007; Lagos et al., 2011; Garcia-Fuster et al., 2012). Therefore, it was interesting to observe whether a potent (in the nM range) MCH_1_ antagonist that was theoretically incapable of penetrating the blood/brain barrier and fairly specific (as a derivative of the natural MCH_1_ agonist MCH) would have an effect *in vivo*, as it was already known to be active i.c.v. (Audinot et al., 2009). S38151 appears to fulfill the task. Indeed, despite a longer stability than unmodified peptides, its stability in blood remains too low to expect further reasonable activity in humans. However, it remains interesting to note that the blockade of the N-terminus of the peptide with a *para*-guanidinobenzoyl moiety may enhance its stability in biological media.

Furthermore, we were unable to detect traces of this pseudopeptide in the brains of i.p.-injected animals. Similarly, its effect could not be correlated with aversion owing to a bad test. Finally, S38151 significantly reduced weight gain in rodent obesity models, and (most importantly) was inactive in our MCH_1_-KO mouse model, strongly suggesting not only the nature of its target but also its potency and selectivity.

The process through which S38151 induces weight loss remains poorly understood at the molecular level. We hypothesize that it acts locally at the adipocyte level. Indeed, in their paper on a small, non-peptide antagonist (SCH-A) of MCH_1_ activity, Kowalski et al. (2006) performed a careful and complete study to describe the mechanism of action of this compound at the adipocyte level. To our knowledge, few peptide MCH agonists or antagonists have been used *in vivo*. Indeed, as reviewed by Bednarek (2007), peptide ligands at MCH_1_ were used *in vivo* by Mashiko et al. (2005) and Shearman et al. (2003) to demonstrate the effects of peptide MCH_1_ antagonists on diet-induced obesity. Both compounds were administered through i.c.v. injection. To our knowledge, none of the reported agonists or antagonists was administered through i.p. injection. The validation of the agonistic nature of MCH, MCH derivatives, or peptides derived from them, has been reported on several occasions regarding feeding behavior, and has always involved administration through i.c.v. injection (Suply et al., 2001) or on their other effects (Chung et al., 2009; Lagos et al., 2009). All of these data have been reviewed by MacNeil and Bednarek (2009).

### Conflict of interest statement

The authors declare that the research was conducted in the absence of any commercial or financial relationships that could be construed as a potential conflict of interest.
